# An Audience Effect in Sooty Mangabey Alarm Calling

**DOI:** 10.3389/fpsyg.2022.816744

**Published:** 2022-02-21

**Authors:** Fredy Quintero, Sonia Touitou, Martina Magris, Klaus Zuberbühler

**Affiliations:** ^1^Department of Comparative Cognition, Institute of Biology, Université de Neuchâtel, Neuchâtel, Switzerland; ^2^Centre Suisse de Recherches Scientifiques, Taï Monkey Project, Abidjan, Côte d’Ivoire; ^3^School of Psychology and Neurosciences, University of St Andrews, St Andrews, United Kingdom

**Keywords:** snake-alarm calls, *Cercocebus atys*, vocal communication, audience effects, intentionality

## Abstract

How does intentional communication evolve? Comparative studies can shed light on the evolutionary history of this relevant feature of human language and its distribution before modern humans. The current animal literature on intentional signaling consists mostly of ape gestural studies with evidence of subjects persisting and elaborating with sometimes arbitrary signals toward a desired outcome. Although vocalizations can also have such imperative qualities, they are typically produced in a functionally fixed manner, as if evolved for a specific purpose. Yet, intentionality can sometimes transpire even in functionally fixed calls, for example, if production is adapted to audience composition. In this study, we carried out field experiments to test whether free-ranging sooty mangabeys adjusted snake alarm call production to their audiences. We found a positive relation between alarm call production and naïve individuals arriving, suggesting that callers attempted to influence their behaviors relative to the snake. Subjects called more with smaller audiences, if they had not heard other calls before, and if socially important individuals were in the area. We concluded that sooty mangabeys alarm call production can be explained as an active attempt to refer to an external event, rather than a mere readout of an internal state.

## Introduction

Studies on animal intentionality are important for a number of reasons, such as for understanding the evolutionary origins of human language and the forces that drive the evolution of communication more generally (Zuberbühler and Gomez, 2018; Krupenye and Call, 2019). Intentionality has been investigated by focusing on specific behavioral markers, such as attention-getters, checking for other’s attentional states with gaze alternation, signal elaboration, as well as flexible or persistent use and response waiting (Bates, 1979; Tomasello et al., 1994; Leavens, 2004; Townsend et al., 2017). Much relevant work has been carried out with great apes, a group of animals that is of special interest for evolutionary questions, due to their phylogenetic closeness to humans. Here, several lines of evidence suggest that individuals can take into account the goals and intentions of others and adjust their own signaling behavior accordingly (Call and Tomasello, 2008; Hare, 2011). For example, studies with captive orangutans, chimpanzees, and bonobos have all shown that individuals are capable of modifying their signal output depending on the attentional state and familiarity of the recipient, with evidence for persistence and elaboration when dealing with unresponsive recipients (Cartmill and Byrne, 2007, 2010; Hobaiter and Byrne, 2014; Genty et al., 2015). A number of great ape field studies have also concluded some vocal behavior also meet criteria for intentionality (e.g., chimpanzees: Crockford et al., 2012; Schel et al., 2013a,b; Hobaiter et al., 2014; bonobos: Genty and Zuberbühler, 2014). The consensus view from this research is that great apes are not only able to perceive and attribute intentions to others, but that they are also able to communicate their own and, if misunderstood or ignored, modify their signaling strategy to achieve the desired goal.

Comparably much less is known from other animals, including other groups of primates. This is problematic because there is a distinct possibility that the underlying cognitive capacities needed for intentionality do not evolve like morphological traits along phylogenetic lines. They might just be mere processing features of large brains or evolve in response to specific social or ecological selection pressures by convergent evolution (Emery and Clayton, 2004). To test whether cognitive mechanisms are part of a species’ phylogenetic history (MacLean et al., 2012), research on monkeys and non-primate species is crucial. Relevant findings come from studies on captive rhesus macaques (*Macaca mulatta*) and tufted capuchins (*Sapajus apella*) which show that subjects can be sensitive to others’ goals and intentions (e.g., Hare et al., 2003; Flombaum and Santos, 2005; Santos et al., 2006; Phillips et al., 2009; Drayton and Santos, 2014). In the wild, there is also evidence showing that some monkey species are able to produce alarm calls with the apparent purpose of influencing others’ behaviors (Zuberbühler, 2018). In one study, wild Thomas langur males continued to produce alarm calls to predator model until every group member had responded with at least one alarm call, as if to ensure that others were aware of the danger (Wich and de Vries, 2006). In another study, wild Diana monkey females continued to alarm call until their own male produced the semantically matching (“correct”) alarm calls, i.e., the predator spotted by the females, in response to which they stopped producing alarm calls (Stephan and Zuberbühler, 2016). Also, playback experiments with blue monkeys showed that males produced significantly more alarm calls to simulations of crowned eagle presence if other group members were closer to the presumed predator than far away (Papworth et al., 2008), further demonstrating some basic audience awareness, but not ruling out explanations based on basic changes in affective states.

Traditionally, studies on animal intentionality have used Dennett’s stages of intentionality as a theoretical framework (Dennett, 1983). Here, a key change is between the second to higher orders of intentionality, which require the ability to attribute mental states during communication. Although Dennett’s framework is appealing, one problem with it is that young children struggle with tasks that require mental state attribution (Wimmer and Perner, 1983; Liddle and Nettle, 2006), that even adults do not always attribute mental states automatically during interactions (Keysar et al., 2003; Apperly et al., 2006) and sometimes even actively avoid them (McClung et al., 2013).

One possibility is that human adults, and certainly pre- and non-linguistic subjects, do not primarily assess others’ behaviors as governed in terms of underlying mental states (beliefs, desires, and intentions), but in terms of rehearsed behavioral or social scripts that allow subjects to make judgments and predictions in how social interactions normally unfold^1^ (Worden, 1996). But even though such cognitively simpler script-based accounts appear to better explain the empirical data, including much of the theory of mind literature, they still rely on intentionality as a basic force of social behavior. For communication signals, Townsend et al. (2017) have proposed three distinct criteria that signal production needs to meet before intentionality can be ascribed to it. Although research on great apes continues to provide evidence for intentional communication (e.g., Gruber and Zuberbühler, 2013; Schel et al., 2013b; Bouchard and Zuberbühler, 2022), to our knowledge there are no comparable studies on free-ranging monkeys designed to tackle the same question.

One way to assess intentional states in animals and other non-linguistic subjects is to present them with private information that is also relevant for others, such as encountering danger. If the subject is able and willing to take another’s intentions (receiver’s presumed knowledge about the danger) into account, it should take active steps to inform its partner, especially if it is still ignorant and likely to endanger itself. One successful paradigm has been to present snake models to lone individuals, without others witnessing the event (Crockford et al., 2012). In chimpanzees, this has led to the conclusion that they are capable of taking into account others’ mental states, due to the fact that they were more likely to call if newly arriving individuals were not aware of the danger (Crockford et al., 2012) and if they were socially important to the caller (Schel et al., 2013b). However, when the same experiment was replicated with sooty mangabeys in Taï Forest (Ivory Coast), callers did not adjust call production to the presence of socially important or referentially unaware partners (Mielke et al., 2019). The reasons have remained unclear, but one possibility is that this represents a cognitive divide between monkeys and apes (Tomasello, 2010), or a mere age effect (mostly juveniles were tested in the monkey study). Appropriate alarm calling requires experience and it is certainly possible that juveniles were unable to process social situations in the same way as adults would (Cheney and Seyfarth, 2007).

Also, field experiments with predator models are prone to authenticity problems (see Zuberbühler and Wittig, 2011) raising the possibility that subjects processed the models differently compared to real snakes. Nevertheless, sooty mangabeys are an ideal species for direct comparisons between monkeys and chimpanzees since they live in the same forest habitat, form similarly sized multi-male, multi-female groups (up to 100), have similar foraging and locomotor habits, although, unlike chimpanzees, they do not have male philopatry and only restricted fission-fusion (Range and Noë, 2002; Aureli et al., 2008).

The goal of this study was to reassess the proposed monkey-ape cognitive divide by focusing only on adult individuals and to revisit the question of primate intentionality more generally. Encounters with dangerous Gaboon and Rhinoceros vipers (*Bitis gabonica*; *Bitis nasicornis*) are common in sooty mangabeys, observed around 3–4 times per week and sometimes several times per day (Range and Fischer, 2004; FQ, unpublished data). This is due to the fact that individuals spend much of their time foraging through the leaf-litter in search for insects and fallen fruits of *Anthonota spp*., *Saccoglotis gabonensis* or *Dialium* spp. (Janmaat et al., 2006; McGraw et al., 2011; Range and Noë, 2002). Sooty mangabeys react very strongly to these two snake species, by giving acoustically distinct snake alarm calls, which can cause others to jump into the lower canopy in order to localize and subsequently approach the snake. Interestingly, adult mangabeys ignore most other species of snakes, including highly poisonous forest cobras (*Naja melanoleuca*) or green mambas (*Dendroaspis viridis*), suggesting that the Gaboon and Rhinoceros vipers had led to lethal accidents and subsequent learning, despite the fact that neither snake is likely a major predator, at least for adult individuals.

To address the previous points, we carried a field experiment in which we presented seven different viper replica models to adult sooty mangabeys (Supplementary Appendix 1), under different social conditions with the following predictions. First, regarding audience size, we predicted that audience size should not influence alarm call production *per se*, since alarm calling is always effective, regardless of the number of listeners. However, the duration of alarm calling should be related to audience size; more specifically, the time it takes for others to arrive at the site. Regarding audience composition, we predicted that adult and experienced callers should be interested in informing mainly snake-ignorant and socially relevant group members.

## Materials and Methods

### Study Site and Subjects

The study was conducted in Taï National Park in South-western Ivory Coast (5°50′N, 7°21′ W). The park is the largest remaining major block of primary forest in West Africa and covers approximately 454,000 ha of continuous forest. The forest is classified as “tropical moist” with a mean annual temperature of 24°C, a mean annual rainfall of 1,875 mm and a distinct dry season in December–January (Whitmore, 1990; Taï Monkey Project Data, 2015). The study area of about 7 km^2^ was situated near the western border of the park, approximately 20 km southeast of the township Taï. The study group’s home range contained a 2-km^2^ core area where groups of several monkey species had been studied since 1991, as part of a long-term research project (McGraw and Zuberbühler, 2007). The sooty mangabey study group has been under constant observation since 1997 and is well habituated to human observers (Range and Noë, 2002; Neumann and Zuberbühler, 2016). Data collection was during group follows from dawn to dusk (7:00 to 17:00 local time) over a period of 24 months in three blocks of time: January to May 2013, August 2013 to July 2014 and January to September 2015. During the study period the group size was around 80 individuals, including 25–30 adult females (> 5 years old), 4 adult males (> 5 years old), 3–8 juvenile females (1–4 years old), 10–18 juvenile males (1–4 years old), and 16–20 unidentified infants (< 1 year old).

### Experimental Design

**FIGURE 1:**
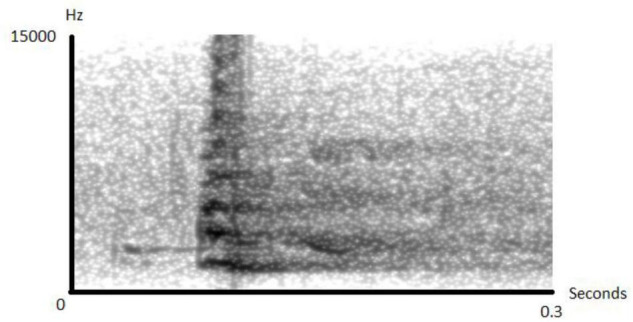
Spectrogram of a snake alarm call given by a female sooty mangabey. When encountering snakes, individuals produce sequences of up to a dozen of such (Spectrogram made in Praat).

When encountering gaboon or rhinoceros vipers, sooty mangabeys respond by giving acoustically distinct “snake” alarms (Figure 1 and Supplementary Audio 2), which attract other group members. Typically, only one adult individual gives alarm calls, suggesting a sort of sentinel function, but the first individual to discover the snake is not always the first caller. Sometimes, up to three additional individuals also produce alarm calls, but these are usually infants or juveniles (FQ unpublished data).

In the experiments, we selectively exposed *N* = 14 adult group members to seven different snake models, authentic replicas of *Bitis gabonica* and *Bitis nasicornis* in various natural positions (Supplementary Appendix 1). Adult subjects were chosen randomly and exposed to the model. Experiments were performed no more than twice per month, with a total of *N* = 22 trials.

For each trial, the audience composition was determined as was the subject’s reaction when discovering the snake. For each encounter, we determined if the discovering individual had already heard a snake alarm given by another group member (to the model).

We then created two data sets. In a first dataset, each snake encounter by an adult individual was entered as one data point, provided (1) no snake alarm call had been produced before the encounter and (2) no individual other than the subject was within 10 m (average maximum visibility) of the model. Alarm calls given within the first 5 min of the subject’s first call were considered part of the same encounter (“ignorance model,” see below; most experiments lasted less than 5 min). In a second data set (“encounter model,” see below), we entered every individual snake encounter as an independent data point regardless of other factors.

### Natural Encounters

During the study period, we recorded *N* = 20 natural snake encounters with mostly Gaboon and Rhinoceros vipers. During the wet season, natural encounters with vipers can happen every single day, but even during drier periods, 1–2 encounters per week are common. Typical encounters happen when an individual finds a viper resting motionless on the ground. Usually, the first individual then responds with a brief startle response followed by an acoustically distinct alarm call (Figure 1). This usually causes other individuals to approach to locate and observe the snake. We never observed anything that could qualify as mobbing behavior, i.e., acoustically or visually conspicuous behavior to drive away the snake or rally other group members. Instead, upon detection, most individuals simply approach and observe the snake in a calm way. In only one encounter the snake was moving, which led some individuals, mostly juveniles, to follow the snake within the lower canopy less than 4 m off the ground with occasional alarm calls. During natural encounters it was nearly impossible to determine the exact moment of snake detection, although it almost always led to large gatherings of monkeys surrounding the snake (see Supplementary Video 1), so we decided to simulate snake encounters using life size replica.

### Experimental Protocol

After selecting a subject, we waited until there was no other individual around 10 m. We then positioned the model so that the subject was the first to discover it. We did this by trying to predict the travel path of the subject and positioning the snake on the forest floor on the anticipated path. Occasionally another individual found the snake first, in which case we made it the subject. During each trial, a first experimenter positioned the snake ahead of the subject’s anticipated travel path and determined the audience composition, by identifying all present and newly arriving group members within the visible range. The second experimenter filmed the subject as it approached the snake, recorded all calls and orally described the event. The two experimenters were accompanied by a field assistant who could assist with the different steps. Individual identification of group members present was essential, which was achieved by having a team of observers. We used a Panasonic Video-Camera SDR-26 to film each trial and Marantz PMD 661 solid state recorder with a Sennheiser MKH 418 microphone to record all calls. From the 22 trials, 6 trials in 2014 were conducted by MM; the rest by FQ. All the data from the videos were coded by FQ. Data extraction from the videos only concerned uncontroversial variables, such as time of arrival, number of calls and number of individuals, but no behavioral or proximity data, which would require interobserver reliability tests. For transparency, we have uploaded all video clips of the different snake encounter trials for inspection^2^.

### Statistical Analyses

During each trial we scored the number of “snake alarms” produced by the subject (numeric: “ncalls”), as the response variable in our models. As predictor variables we included (a) the subject’s sex (binary: “sex”), (b) the time interval between the subject’s first alarm call and the arrival of the first audience member at the snake location (numeric: “time1starrival”), (c) the number of individuals within 10 m when the subject detected the snake (numeric: “neighbors”), (d) whether the subject discovered the snake first (binary: “ffinder”), (e) the presence of socially important individuals (binary: “friend”; defined by a DSI score > 1; Silk et al., 2013). For calculating the DSI we used the following behaviors as variables: “approach,” “inspection,” “presenting groom,” “contact,” “groom,” “handle baby” and “hug” (Supplementary Table 1); (f) the social status of the subject (numeric: “rank”; determined by its Elo-rating score; Neumann et al., 2011), (g) the number of individuals that arrived at snake location the first alarm call (numeric: “nbarrivals”), (h) time the subject stayed within 10 m of the snake after producing the first call (numeric: “findertime”), (i) the number of calls heard before arriving at the snake location (numeric: “ocbefore”). We did not use a predictor for call secession in response to detection as sooty mangabeys do not typically stop calling when others arrive. Nonetheless, if there would be a pattern in others arriving and call cessation this would be reflected in the “time1starrival” variable. We then created two models to assess the factors driving snake alarm calling in sooty mangabeys.

### The “Encounter” Model

We used generalized linear mixed models (GLMM) with a Poisson error structure to test variation in call production for every individual that encountered the snake, regardless of the previous alarm call history. To this effect, every individual that approached the snake was entered as a data point, regardless of whether any other individual had produced a snake alarm call before or after, with the same factors entered as mentioned above, except for the ones only relevant for the caller (“findertime,” “nbarrivals,” and “time1starrival”). With this model, we addressed whether audience size (N neighbors) affected call production, provided there were previous snake alarms (neighbors * ocbefore). Second, we addressed whether audience composition (socially important individual present) affected call production, provided there were previous snake alarm calls (friend * ocbefore). Third, we addressed whether high-status individuals were more likely to vocalize than low-status individuals, provided they detected the snake (rank * ffinder). Finally, we addressed whether the audience composition (socially important individual present) affected call production, provided they detected the snake (snake * ffinder). For all models we included random intercepts for the focal subject ID.

We then built an “informed null model,” which comprised all fixed terms except those that included the main predictors. The random structure was identical to the full model. We then compared these models with a likelihood ratio test (Dobson, 2002). If the comparison of full and null model revealed significance, we explored the full model with regards to our predictors of interest (i.e., those that were in the full but not in the null model).

### The “Ignorance” Model

We used linear mixed models (LMM) to determine the factors that affected the number of alarm calls produced from the caller’s perspective. Every individual that found the snake and had not heard an alarm call before was entered as an independent data point. All the factors above mentioned were included except for the number of calls heard before arriving at the location (“ocbefore”). We included random intercepts for focal subject ID. We then conducted a model selection procedure from the global model to determine the factors that best predicted call production. Models were ordered by the value of the Akaike information criterion, with the lowest on top. The validity of the best model was then checked with a Shapiro-Wilk test.

We used R version 4.0.3 (R Core Team, 2020) for the analyses above mentioned, with the glmer and lmer functions, “lme4” package (Bates et al., 2015) for the GLMMs and LMMs. We also used the dredge function, “MuMIn” package (Barton, 2018) for the model selection.

### Ethical Note

The methods used in this study are in line with the Animal Behavior Society Guidelines for the Use of Animals in Research. We used non-invasive methods for the observation of the subjects in their natural habitat. The animals were identified by physical features like scars, body size and shape, and they were all habituated to human observers. The experiments simulated a natural event and did not interfere with the animals’ normal daily routine. Research permission and ethical clearance were granted by the Ministère de la Recherche Scientifique et Technique de Côte d’Ivoire.

## Results

### Experimental Encounters

We carried out *N* = 22 trials during which we presented seven snake models to *N* = 27 individuals unaware of the snake (adult females: *N* = 24; adult males: *N* = 1; juvenile males: *N* = 2). Subjects alarm called in 21 of 27 encounters (77.8%). We found that, every time a subject alarm called, other group members responded by approaching and looking for the snake, generally silently. In addition, juveniles often grabbed and smelled leaves near the snake in order to smell them, suggesting that there is an olfactory component relevant to snake encounters. Silent encounters without alarm calls were also considered in both models.

### The “Encounter” Model

**FIGURE 2:**
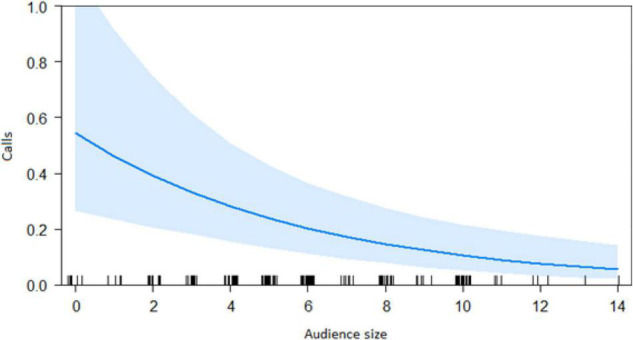
Effect of audience size on the number of calls when finding the snake (Audience size = Number of individuals around 10 m of the subject). Calls: Proportion of alarm calls given by the subject.

**FIGURE 3:**
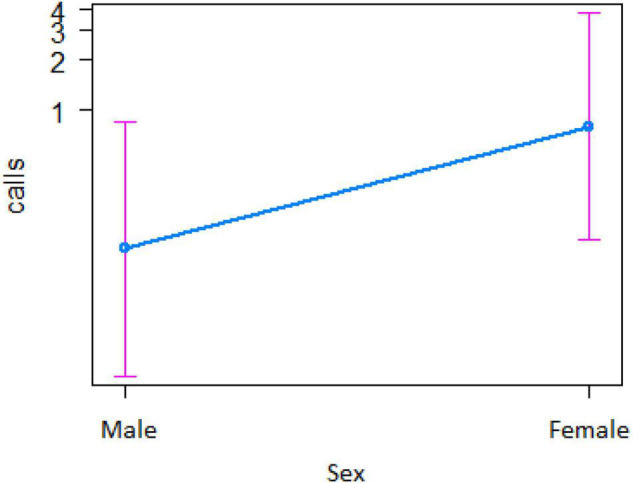
Effect of caller’s sex on the number of calls when finding the snake (*Y*-axis in log scale). Sample size 132 adult females, 8 adult males, and 10 juvenile males.

**FIGURE 4:**
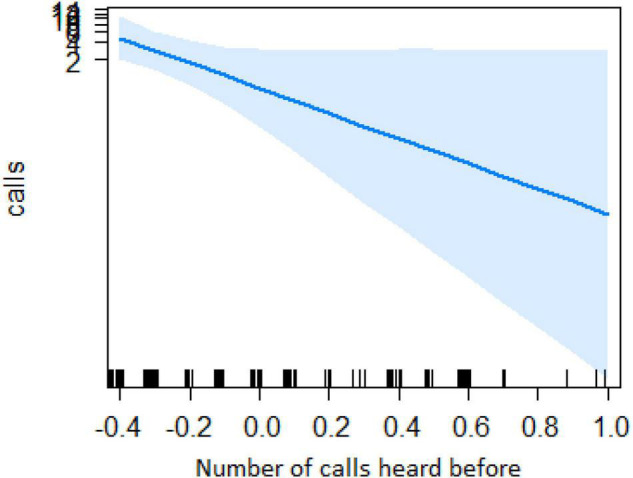
Effect of the number of calls heard before on the number of calls when finding the snake (*Y*-axis in log scale). X-axis data has been transformed so it could be better visualized.

**FIGURE 5:**
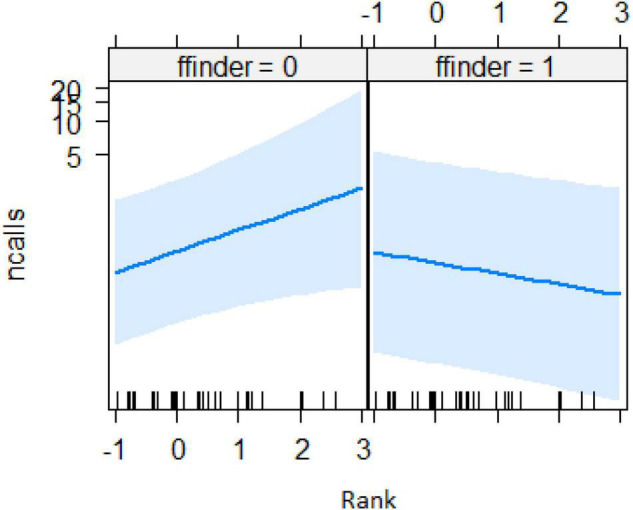
Effect of caller’s rank when being the first to find the snake (*Y*-axis in log scale). Rank data has also been transformed so it could be better visualized.

In the encounter model, we investigated which variables best explained the number of calls produced when individuals found the snake. We registered *N* = 150 snake encounters when considering all the individuals that approached the snake regardless of awareness. We found that the number of snake alarm calls (a) decreased with increasing numbers of individuals already present (beta ± SE = −0.8541 ± 0.5, *p* = 0.02410; Figure 2 and Table 1), (b) decreased with increasing caller social status (beta ± SE = 0.451 ± 0.21, *p* = 0.037; Table 1), (c) increased if the callers were female (beta ± SE = 1.69 ± 0.58, *p* = 0.0035; Figure 3 and Table 1), and (d) decreased with a lower number of calls before (beta ± SE = −9.997 ± 4.96, *p* = 0.044; Figure 4 and Table 1). Two interactions were linked to significant increases in call production: discoverer * low ranking (beta ± SE = −0.6720 ± 0.23, *p* = 0.00451; Figure 5 and Table 1), no prior alarm calls * socially important individual in the audience (beta ± SE = 9.41 ± 5, *p* = 0.059; Table 1). The full model was significantly different from the null model (χ^2^ = 158.36, df = 3, *p* = 0.000).

**TABLE 1:** Results of the GLMM for the number of alarm calls when individuals find a snake regardless of whether there has been a previous alarm call.

Variables	Estimate	SE	*Z*	Pr (> | z|)
(Intercept)	−2.791	0.716	−3.895	0.000
Rank	−0.166	0.258	−0.644	**0.037**
Sex	0.667	0.719	0.928	**0.003**
Friend	0.492	0.230	2.134	0.248
First finder	2.520	0.326	7.723	0.285
Neighbors	−0.535	0.139	−3.831	**0.024**
Call heard before	−0.239	0.076	−3.154	**0.044**
Call after	−0.348	0.215	−1.612	0.106
Ffinder:Rank	−0.672	0.236	−2.840	**0.004**
Ffinder:Friend	2.001	1.141	1.754	0.079
Friend:Call heard before	9.413	5.002	1.882	**0.059**
Neighbors:C. heard before	−1.493	0.981	−1.522	0.128

*The bold values are the significant values from “The Encounter model”.*

### The “Ignorance” Model

**FIGURE 6:**
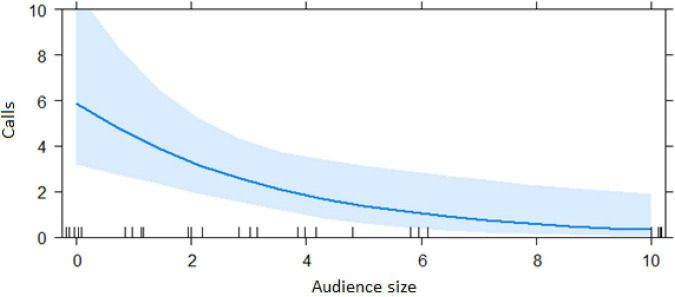
Effect of audience size on the number of alarm calls by ignorant individuals encountering the snake (Audience size = Number of individuals within 10 m of the subject). Calls: Number of alarm calls given by the subject.

**FIGURE 7:**
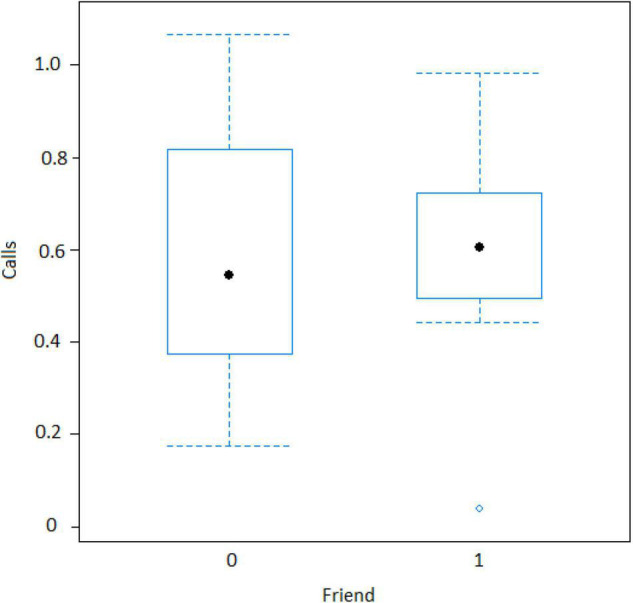
Effect of audience composition on the numbers of calls when an ignorant individual finds the snake (Friend = Socially important individual present around 10 m of the subject). Calls: Proportion of alarm calls given by the subject.

**FIGURE 8:**
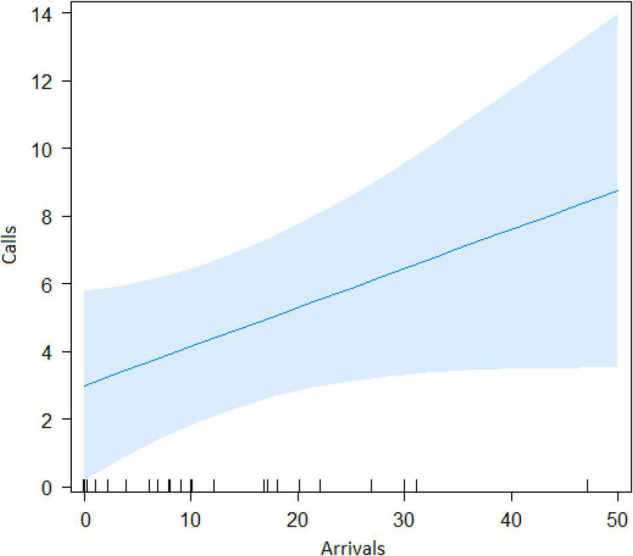
Consequence of alarm calling. Effect of the number of calls given on the number of individuals that arrive at the snake position.

The best model for ignorant individuals detecting the snake (AIC = 65.7; Table 2) showed that the number of snake alarm calls (a) decreased with increasing numbers of individuals already present (beta ± SE = −0.735 ± 0.17, *p* = 0.00062; Figure 6 and Table 3), (b) increased with the number of socially important individuals already present (beta ± SE = 0.611 ± 0.23, *p* = 0.023; Figure 7 and Table 3) and (c) predicted how many individuals would arrive to see the snake (beta ± SE = 0.285 ± 0.05, *p* = 0.0001; Figure 8 and Table 3). A Shapiro-Wilk test indicated that the data were normally distributed (W = 0.98387, *p*-value = 0.9374).

**TABLE 2:** Model selection table for the LMM for the number of alarm calls of ignorant individuals encountering the snake.

Intrc	Friend	Sex	Neighbors	Nbarrivals	Time1starrival	Df	logLik	AICc	Delta	Pr (> | z|)
101	+		−0.7358	0.285		6	−24.7	65.7	0.00	0.268
201		+		0.290	0.1558	6	−25.2	66.6	0.87	0.174
105		+	−0.4145	0.246		6	−25.2	66.7	0.90	0.171
109	+	+	−0.6806	0.281		7	−23.4	66.8	1.00	0.162
97			−0.4591	0.2451		5	−27.2	67.3	1.56	0.123
73		+		0.228		5	−27.4	67.7	1.94	0.102

**TABLE 3:** Results of the best LMM for the number of alarm calls of ignorant individuals encountering the snake.

Variables	Estimate	SE	df	*t* value	Pr (> | z|)
(Intercept)	1.549	0.340	20.6	4.55	0.0001
Friend	0.611	0.239	13.47	2.55	0.0234
Neighbors	−0.735	0.173	16.2	−4.23	0.0006
Number of Arrivals	0.285	0.056	14.3	5.01	0.0001

## Discussion

We carried out field experiments with free-ranging monkeys in Tai National Park, Ivory Coast, to address the question of whether callers were capable of warning each other of the presence of dangerous snakes. Using live-sized realistic replicas of two highly dangerous vipers we managed to elicit responses from sooty mangabeys that largely matched natural observations. Individuals that first discovered the model typically alarm called and stayed with the snake until other group members arrived. In order to address questions of whether these calls qualified as intentional signals, we specifically looked at how audience-related variables impacted on call production. To this effect we tested two separate models, one considering data from any individual that discovered the snake and another one considering only encounters by individuals that were ignorant about the snake at the moment of detection.

Both models revealed that audience was an important factor influencing the number of calls when encountering snakes. Generally, subjects increased their calling efforts with decreasing audience sizes (Figures 2, 6). For ignorant callers (callers unaware of the snake presence that find it before others), higher number of calls were related with the presence of socially important individuals (Figure 7), and this also led to a higher number of arrivals (Figure 8). We also found that having heard an alarm call before led to significantly fewer alarm calls than if no call was given before. These findings would be consistent with the interpretation that, similar to chimpanzees, sooty mangabeys would have some concerns for others in these potentially dangerous situations and alarm called specifically if the benefits for others were high (if most of group members were still absent and if friends were exposed to danger). But in sooty mangabeys this could also be explained by affective or physiological changes related to variation in group size and audience composition.

Sooty mangabey snake alarm calls can be heard over distances of at least 100 m, suggesting that most group members will be informed if an individual calls to a snake. It is therefore somewhat surprising that audience size had an effect on alarm calling at all. As explained earlier, we never observed mobbing behavior in sooty mangabeys, neither in natural nor during experimental snake encounters. Gaboon vipers are highly static snakes, unlikely to move in response to agitated monkey display behavior, suggesting that mobbing would be ineffective as an anti-predator strategy. One possibility is that callers were not just interested in informing others about the snake, but that calling also served to reveal its location. Clearly, recipients will benefit mostly if they know the exact location of the danger, which is only possible following personal inspection. This is supported by the fact that the number of arrivals increased with a higher number of alarm calls. Further research is needed regarding the behavior of the caller right after finding the snake.

We found no evidence that the timing of others’ arrivals influenced the subject’s calling behavior (Table 2), suggesting that it is not paramount for callers to make sure that all group members have seen the snake (in contrast to, for example, results from Thomas langurs; Wich and de Vries, 2006). During experiments and real encounters, we observed that only a fraction of the group approached the snake, whereas many other group members appear to ignore the event. In contrast to species living in small groups, such as Thomas langurs, it may simply not be feasible for callers to continue calling until the entire group has witnessed the snake, especially as this is unlikely to happen anyway. As mentioned, Gaboon and Rhinoceros vipers are not primary predators, despite being highly dangerous, suggesting that knowing their location is the main requirement to remain safe. Knowing which general area to avoid is therefore enough, while visually locating the snake is only needed if the subject wants to use that area, for example for foraging.

Our work partly replicates a previous study on sooty mangabeys (Mielke et al., 2019) by showing that subjects were less likely to call if they heard a call before and if they were with large audiences. This reinforces the idea that it is important for callers to ensure that as many individuals as possible know the snake location. Also, snake alarm calls are usually produced by individuals near the snake, suggesting that these individuals subsequently act as visual beacons to mark the snake’s location.

However, in our study we also found both caller and audience effects: (1) the four adult males never called to snakes, (2) lower ranking individuals were more likely to call, and (3) alarm calling was more important when socially important individuals were in the audience. In mangabey groups, adult males are socially peripheral, whereas adult females form the social core of the group. Perhaps, unsurprisingly they were thus also more likely to give alarm calls to snakes (Figure 3), perhaps to provide social learning opportunities to their more vulnerable offspring (Seyfarth and Cheney, 2010). The fact that the presence of “friends” was also associated with higher alarm calling rates (Table 3 and Figure 7), suggests that grooming could be traded with warning for Gaboon or Rhinoceros vipers.

Previous work with wild Thomas langurs has shown that callers continue until every single individual has encountered the predator, while female Diana monkeys will not stop calling to a leopard until their males have produced the same calls (Wich and de Vries, 2006; Stephan and Zuberbühler, 2016), suggesting that alarm calling is tied to underlying intentions to inform others. In our study, we did not find such pattern but calling was most common in individuals who found the snake, provided no call had been produced before (Table 1) and with key individuals in the audience, to our knowledge a first such demonstration in free-ranging monkeys. The first criteria for intentionality is goal directed behavior that can be measured as persistence. Nonetheless, there are significant trends where sooty mangabeys call more when there are less individuals and the more they call, the more that other individuals are recruited. Regarding the three criteria for intentional communication, put forward by Townsend et al. (2017), we can state the following: (1) Regarding “goal directedness,” alarm calling was about detecting the snake and, presumably, ensuring that others were made aware of its location. Although we did not observe any obvious signs of persistence (i.e., callers monitoring exactly whether or when others have located the snake) we found that mangabeys called more when fewer individuals were present and that the more they called the more individuals arrived. (2) Regarding “recipient-directedness” our data resemble findings in chimpanzees who produce food calls and snake alarms preferentially in the presence of socially important individuals, i.e., friends and high-ranking group members (Schel et al., 2013a,b), a pattern we also found (Table 3 and Figure 7). In contrast to chimpanzees, however, we never observed an individual re-starting alarm calling with the arrival of a new individual. Whether or not these patterns could be explained with more basic changes in physiological states or arousal, rather than an intentional stance, would have to be further investigated. (3) The third criteria for intentionality states that receivers must regularly respond in a way that is in line with the signaler’s presumed intentions. Although we did not address this requirement directly, there was not a single occasion when sooty mangabeys called and no individuals arrived to locate the snake, suggesting that receivers responded in line with the caller’s expectations.

In conclusion, sooty mangabey snake alarm calling is driven by several factors related to the caller and affects other group members who approach the caller to then try to locate the danger. The patterns are not in line with a more traditional notion of animal calls as hardwired reflexive responses to specific stimuli, but appear to involve assessments of both ecological and social variables in ways that meet criteria of intentional signaling.

## Data Availability Statement

The raw data supporting the conclusions of this article will be made available by the authors, without undue reservation.

## Ethics Statement

The animal study was reviewed and approved by Ministère de la Recherche Scientifique et Technologique de Côte d’Ivoire.

## Author Contributions

FQ and KZ: study design, interpretation, and drafting the article. ST, MM, and FQ: data collection. FQ: statistical analysis. KZ: provision of necessary tools and resources. All authors read and approved the final manuscript.

## Conflict of Interest

The authors declare that the research was conducted in the absence of any commercial or financial relationships that could be construed as a potential conflict of interest.

## Publisher’s Note

All claims expressed in this article are solely those of the authors and do not necessarily represent those of their affiliated organizations, or those of the publisher, the editors and the reviewers. Any product that may be evaluated in this article, or claim that may be made by its manufacturer, is not guaranteed or endorsed by the publisher.

## Footnotes

1 Taylor, D., Alexander, C., Gönül, G., Clément, F., Zuberbühler, K., and Glock, H. J. (submitted). Reading minds or reading scripts? De-intellectualising theory of mind. *Proc. B.*

2
https://figshare.com/s/82b6989a71e75a57e523
