# Supplementary material for: An Audience Effect in Sooty Mangabey Alarm Calling
Source: Front Psychol. 2022 Feb 21;13:816744. doi: 10.3389/fpsyg.2022.816744 (PMC8899475; doi:10.3389/fpsyg.2022.816744)
Supplement: Supplementary file 3 [file Data_Sheet_1.docx]

Appendices

Figure S1


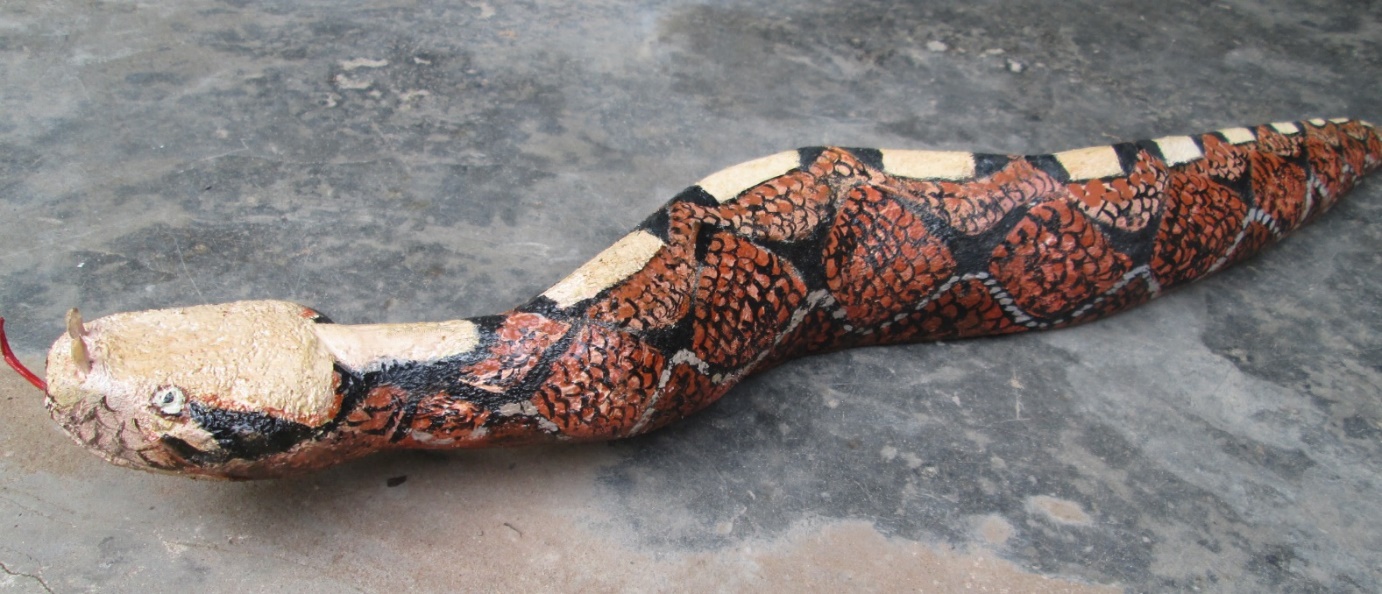


The snake models were made by the local artist Djobely et Artable, who crafted them from wood and painted them to look as closest as possible to real Gaboon and Rhinoceros Vipers. Seven different snake models were made in different positions resembling the same postures that real snakes show in the wild.

**Table S1.**  Ethogram of sooty mangabeys of Taï National Park, Ivory Coast. Adapted from Range & Noë (2002).

|  | **Abbreviation** | **Behaviour** | **Definition** |
| --- | --- | --- | --- |
| Affiliative Behaviours | Co | Contact | two individuals are in contact sitting usually and don't really interact with each other |
|  | Hu | Hug | two individuals are hugging each other (usually around the shoulders or neck) |
|  | Hb | handle baby | individual is manipulating the baby |
|  | Ca | carry baby without stress | individual is carrying a baby to move around (usually on the stomach but sometimes on the back) |
|  | Mtm | mouth to mouth | individual is smelling a partner's mouth and vice versa |
|  | Lm | lips smacking | individual is making noise by clapping its lips (usually while grooming or approaching a subordinate) |
|  | Pl | play (general) | individual is playing (no description) |
|  | Gr | Groom | individual is grooming a partner |
|  | Pr | prensenting groom | individual is presenting a part of its body to be groomed (usually elbow, head or back) |
|  | nas | non-agonistic supplant | individual is approaching another animal who is occupying a resource and replaces that individual without overt aggression |
|  | Ap | approach | individual is approaching another animal (r<2m) |
|  | To | Touch | individual touch a partner in a gentle way, hand on shoulder |
|  | Ins | Inspection | Individual inspects another individual sexual parts. |
|  | Fo | Follow | individual is following another animal |
|  | Le | Leave | individual is leaving the proximity of another (perimeter of three meters) |
|  | Ipl | invitation to play | initiation of play (show teeth, look upside down, quick touch before darting and jumping) |
| Agonistic Behaviours | Fi | Fight | two partners stand up and slap each other |
|  | asf | attempt stealing food | individual is trying to steal another's food |
|  | akb | attempt kidnapping baby | individual is trying to steal a mother's baby |
|  | Bt | bared teeth | individual is showing its teeth (usually while approaching a dominant) |
|  | Av | avoidance | individual is avoiding another individual who is approching it |
|  | Jp | jump aside | individual jump aside to avoid a partner, an aggression, something scary |
|  | Cr | crawl on the ground | individual is lying on the ground while looking at its aggressor |
|  | Fl | Flee | individual is fleeing an aggressor |
|  | Lo | look around for support | individual is searching around him for support while it is threatened by another |
|  | cas | carry baby with stress | individual is carrying a baby to escape a stressful situation for itself or for the baby (infanticide attacks) |
|  | St | Stare | individual is threatening (look / go forward) |
|  | Stl | stare and lunge | individual is threatening and half attacking (go forward / backward) |
|  | Gp | Grip | individual is gripping the other's fur |
|  | Bi | Bite | individual is bitting |
|  | Ch | Chase | individual is chasing another |
|  | Sf | stealing food | individual is stealing another's food |
|  | Tp | take place | individual takes another's place |
|  | Kb | kidnapping baby | individual managed to steal a baby and refuse to hand it back to its mother |
|  | Ign | ignore / turn back | individual ignore a partner's sollicitation (for grooming, mating, nursing, etc) and even can turn its back |
|  | Sa | support aggressor | individual supports the aggressor in a conflict |
|  | Sv | support victim | individual supports the victim in a conflict |
